# Learning and fine-tuning a generic value-selection heuristic inside a constraint programming solver

**DOI:** 10.1007/s10601-024-09377-4

**Published:** 2024-11-23

**Authors:** Tom Marty, Léo Boisvert, Tristan François, Pierre Tessier, Louis Gautier, Louis-Martin Rousseau, Quentin Cappart

**Affiliations:** 1 Polytechnique Montréal, Montreal, Canada; 2 Ecole Polytechnique, Palaiseau, France; 3 MILA, Quebec Institute of Learning Algorithms, Montreal, Canada

**Keywords:** Constraint programming, Branching heuristics, Reinforcement learning

## Abstract

Constraint programming is known for being an efficient approach to solving combinatorial problems. Important design choices in a solver are the *branching heuristics*, designed to lead the search to the best solutions in a minimum amount of time. However, developing these heuristics is a time-consuming process that requires problem-specific expertise. This observation has motivated many efforts to use machine learning to automatically learn efficient heuristics without expert intervention. Although several generic *variable-selection heuristics* are available in the literature, the options for *value-selection heuristics* are more scarce. We propose to tackle this issue by introducing a generic learning procedure that can be used to obtain a value-selection heuristic inside a constraint programming solver. This has been achieved thanks to the combination of a *deep Q-learning* algorithm, a tailored *reward signal*, and a *heterogeneous graph neural network*. Experiments on *graph coloring*, *maximum independent set*, *maximum cut*, and *minimum vertex cover* problems show that this framework competes with the well-known impact-based and activity-based search heuristics and can find solutions close to optimality without requiring a large number of backtracks. Additionally, we observe that fine-tuning a model with a different problem class can accelerate the learning process.

## Introduction

Combinatorial optimization has countless industrial applications, such as scheduling, routing, or finance. Unfortunately, most of these problems are NP-hard and, thereby, challenging to solve efficiently. It is why finding good solutions has motivated intense research efforts for many years. Traditional methods for tackling them are somehow based on a *search procedure*: A clever enumeration of the solution space is performed to find a feasible and possibly optimal solution. Among these methods, *constraint programming* (CP) is an exact procedure. It constitutes a popular approach as it offers the possibility to find the optimal solution or good feasible approximations by stopping the search early. An additional asset is its declarative paradigm in modeling, which makes the technology easier for the end-user to grasp. Introducing solver-agnostic modeling languages, such as MiniZinc [1] has greatly facilitated this aspect. Aligned with this goal, the propagation engine inside a CP solver is mostly hidden from the end-user. However, ensuring a generic search procedure is trickier as non-trivial heuristics must be designed to make the solving process efficient for an arbitrary problem. That being said, generic *variable-selection* and *value-selection* heuristics have been successfully designed. Notable examples are *impact-based search* [2] or *activity-based search* [3], but they require computationally intensive initialization and yield poor performance at the beginning of the search. Contrastingly, [4] introduce a generic heuristic designed to guarantee that the initial solution found is of relatively high quality. However, its relevance diminishes as the search progresses. This makes these methods not always appropriate for general use. As a concrete example, the current version of MiniZinc^1^ does not propose generic value-selection heuristics, except *in(out)domain* or *impact-based search*. In practice, heuristics are often designed thanks to problem-specific expert knowledge, which is often out of reach for end-users that do not have a solid background in artificial intelligence.

In another context, *machine learning* (ML) has been recently considered for automating the design of heuristics, both in constraint programming [5, 6], mixed-integer programming [7–9], column generation [10, 11], decision diagrams [12, 13], or SAT solving [14, 15]. Specifically, *reinforcement learning* (RL) [16] or *imitation learning* [17] approaches, often combined with *deep learning* [18], have gained special attention. Although this idea seems appealing, this is not an easy task to achieve in practice as several technical considerations must be taken into account in order to ensure both the efficiency and the genericity of the approach. In constraint programming, we identified three questions to resolve when learning a generic branching heuristic inside a solver. They are as follows:

1. *How to train the machine learning model?* An intuitive way is to leverage an RL agent that would explore the tree search by making branching decisions and rewarding it based on the quality of the solution found on a terminal node. This would typically be done with a *depth-first search* traversal of the tree for getting a certificate of optimality. However, as pointed out by several authors [19, 20], the backtracking operations inside a solver raise difficulties when formalizing the task as a Markov decision process and may require redefining it. Besides, this training scheme intensifies the *credit assignment problem* [21], ubiquitous in reinforcement learning.
2. *How to evaluate the quality of a value selection?* A core component of an RL environment is the *reward function*, which gives a score to each decision performed. The end goal for the agent is to perform a sequence of decisions leading to the best-accumulated sum of rewards. In our case, an intuitive solution would be to reward the agent according to the quality of the solution found. However, this information is only available at terminal nodes, and only a zero reward is provided in branching nodes. This is related to the *sparse reward* problematic, which is known to complicate the training process.
3. *How to learn from a CP model?* This question relates to the type of architecture that can obtain a value-selection heuristic from a search node (i.e., a partially solved CP model). A promising direction has been proposed by [8] for binary mixed-integer programs. They introduced a bipartite graph linking variables and constraints (i.e., the two types of nodes) when a variable is involved in a given constraint. The subsequent architecture is a *heterogeneous graph neural network*. However, this encoding is not directly applicable in constraint programming, as a CP model generally involves non-binary variables and combinatorial constraints. This has been partially addressed by [22], who introduced a tripartite graph where variables, values, and constraints are specific types of nodes. However, this approach lacks genericity as the method requires retraining when the number of variables changes. Another representation is proposed by [23], but the architecture is used as a stand-alone heuristic and is not integrated into a CP solver. Another architecture was proposed by [24]. The core concept involves representing each constraint with its abstract syntax tree and merging similar elements, such as a single variable occurring across multiple constraints. A major limitation of this method, however, is the substantial size of the resulting graph.

Answering such questions is still an open challenge in the research community. This paper proposes to progress in this direction. It introduces a generic learning procedure that can be used to obtain a value-selection heuristic from a constraint programming model given as input. The approach has been designed to be generic in that it can be used for any CP model given as input. In practice, a specific way to extract *features* from a constraint should be designed for any available constraint, but this has to be done only once per constraint type. We limit our experiments to four combinatorial optimization problems, namely *graph coloring*, *maximum independent set*, *maximum cut*, and *minimum vertex cover*. Specifically, we propose three main contributions, each dedicated to addressing one of the aforementioned difficulties. They are as follows: (1) a learning procedure, based on restarts, for training a reinforcement learning agent directly inside a CP solver, (2) a reward function able to assign non-zero intermediate rewards based on the propagation that has been carried out during the search, and (3) a neural architecture based on a tripartite graph representation and a heterogeneous graph neural network. Experimental results show that combining these three ideas enables the search of a CP solver to find good solutions without requiring many backtracks and competes with the well-known impact-based and activity-based search heuristics. Additionally, we observe that fine-tuning a model with a different problem class can accelerate the learning process.

This paper is an extended version of a paper accepted at the *29th International Conference on Principles and Practice of Constraint Programming* (CP 2023) in Toronto, Canada [25]. The main improvements in this version are: (1) additional recent references, (2) a detailed description of the technical background, (3) formalization and description of the training and solving algorithms, (4) experiments on the *maximum cut* problem with 80 nodes, (5) a new case study on the *minimum vertex cover*, (6) an analysis of the framework’s generalization ability, (7) fine-tuning experiments for instances of a different problem class, and (8) identification of two new challenges for future work. The paper is structured as follows. The next section presents other approaches related to our contribution. Then, Section 3 introduces succinctly technical background on reinforcement learning and graph neural networks. The core contributions are then presented in Section 4 and the resulting CP algorithm is provided in Section 5. Finally, Section 6 provides experimental results and closes with a discussion of the results.

## Related work

Bengio et al. [26] identified three ways to leverage machine learning for combinatorial optimization. First, *end-to-end* learning aims to solve the problem only with a trained ML model. This has been, for instance, extensively considered for the traveling salesman problem [27–29] and for other combinatorial problems [30, 31]. However, such an approach does not guarantee the optimality of the solution obtained. Second, *learning to configure* is dedicated to providing insights to a solver before its execution. This can be, for instance, the decision to linearize the problem in the context of quadratic programs [32] or to learn when a decomposition is appropriate [33]. It has also been used in a learning-based approach for optimizing the neighborhood size in the local branching heuristic for mixed-integer linear programming  [34]. This approach is also referred to as parameter tuning [35]. We refer to the initial survey for extended information about these two families of approaches. Third, *learning within a search procedure* uses machine learning within the solver. Our contribution belongs to this last category of methods. Although the idea of combining learning and searching for solving combinatorial optimization problems was already discussed in the nineties [36], it has re-emerged recently with the rise of deep learning. Most combinatorial optimization solvers are based on *branch-and-bound* and *backtracking*. In this context, ML is often used with branching rules to follow. *Imitation learning* [17, 37] has been for instance used to replicate the expensive *strong branching* strategy for mixed-integer programming solvers [7, 8, 38]. One limitation of imitation learning is that the performances are bounded by the performance of the imitated strategy, which remains heuristic and perfectible [39]. This opens the door for RL approaches [11, 28, 40] that have the guarantee to find the best branching strategy eventually [41]. A branching strategy can be split into two challenging decisions, *variable-selection* and *value-selection*. Reinforcement learning approaches have been considered for both of them.

Concerning the learning for selecting the next variable to branch on, [20] proposed to combine a *double deep Q-network* algorithm [42] with a graph neural network for carrying out this task. The approach is trained to minimize the expected number of nodes to reach a leaf node using the *first-fail principle*. Although this is a good proxy for pruning a maximum of infeasible solutions for a constraint satisfaction problem, it does not extend naturally to optimization variants, for which one should consider a trade-off between the quality of the solution found and the number of nodes required to reach that solution. Similarly, [15] leveraged a graph neural network to initialize a variable-selection heuristic for *Chuffed*, a hybrid CP-SAT solver. In an online setting, [6] also proposed to learn variable ordering heuristics where training time is included in the total solving time. Bandit-based learning approaches were also considered to automatically select search heuristics [43–45].

For the value-selection heuristic, [46] introduced a scoring function which gives a score indicating how good an assignation is, given the current domain. A training phase is carried out in a supervised manner to learn this scoring function. [5] proposed to train a model with reinforcement learning outside the CP solver and to integrate the agent, once trained, subsequently in the solver. This has been achieved by reaping the benefits of a dynamic programming formulation of a combinatorial problem. An important limitation of this work is that no information related to the CP solver, such as the propagation achieved on a node, can be used to drive the decision. [22] mitigated this issue by carrying out the learning inside the solver. The model is trained to find the optimal solution and to prove it with the least number of explored search nodes. However, this goal is disconnected from finding the best solution as quickly as possible and is practically hard to achieve, even with a good heuristic. A more realistic goal is to find a good solution quickly without closing the search. This is how the contribution of this paper is positioned.

We want to point out that learning *how to branch* is not the only way to leverage ML inside a combinatorial optimization solver. Related works have also been proposed on learning tight optimization bounds [12, 47] or for accelerating column generation approaches [10]. A recurrent design choice is an architecture based on graph neural networks and a training with reinforcement learning. We refer to the following surveys for more information about combinatorial optimization with graph neural networks [48] and with reinforcement learning [41].

## Technical background

This section introduces the required background on reinforcement learning and graph neural networks to grasp the technical aspects of the paper.

### Reinforcement learning

Let $\langle S,A,T,R \rangle$ be a 4-tuple representing a *Markov decision process* where *S* is the set of states in the environment, *A* is the set of actions that the agent can do, $T: S \times A \rightarrow S$ is a transition function leading the agent from one state to another, given the action taken, and $R: S \times A \rightarrow \mathbb {R}$ is a reward function of taking an action from a specific state. The sequence $[s_1,\dots ,s_T]$ from the initial state ($s_1$) of an agent towards a terminal state ($s_T$) is referred to as an *episode*. The returned reward within a partial episode $[s_t,\dots ,s_T]$ can be formalized as follows: $G_t = \sum _{i=t}^T \gamma ^{i - t} R(s_i,a_i)$, where $\gamma \in [0,1]$ is the discounting factor. The agent is governed by a policy $\pi : S \rightarrow A$, which indicates the action that must be taken on a given state. The agent’s goal is to find the policy that will lead it to maximize the accumulated reward until a terminal state is reached. The core idea of reinforcement learning is to determine this policy by letting the agent interact with the environment and increasing the probability of taking action if it leads to high subsequent rewards. There are a plethora of reinforcement learning algorithms dedicated to this task, such as *trust region policy optimization* [49] or *soft actor-critic* [50]. We refer to *SpinningUp* website for explanations of the main algorithms [51].

This section presents the core principles of *deep Q-learning* [52], which is the algorithm used in this paper. The idea is to compute an *action-value* function $Q^{\pi }(s_t, a_t) = G_t$. Intuitively, this function gives the accumulated reward that the agent will obtain when performing the action *a* at state *s* while subsequently following a policy $\pi$. The output of this function for a specific action is referred to as a *Q-value*. Provided that the action-value function can be computed exactly, the optimal policy $\pi ^\star$ turns out to be simply the selection of the action having the highest *Q*-value on a specific state: $\pi ^* = \textsf{argmax}_\pi Q^{\pi }(s,a), \ \forall (s,a) \in (S,A)$. Although the exact computation of *Q*-values can theoretically be performed, a specific value must be computed for each pair of states and actions, which is not tractable for realistic situations. It is why a tremendous amount of work has been carried out to approximate accurately and efficiently *Q*-values. Among them, deep *Q*-learning aims to provide a neural estimator $\widehat{Q}(s,a,\theta ) \approx Q(s,a)$, where $\theta$ is a tensor of parameters that must be learned during a training phase. This algorithm is commonly enriched with other mechanisms dedicated to speed-up or stabilizing the training process, such as the *double deep Q-network* variant [42] or *prioritized experience replay* [53]. Concerning the neural architecture, we opted for a graph neural network, which is explained in the next section.

In this paper, we refine the standard deep *Q*-learning framework through a series of modifications designed to enhance the stability and efficiency of the learning process. A main element of our methodology is the adoption of the *Huber loss* function, as introduced by [54], for the update of *Q*-values. This approach is further complemented by the incorporation of multi-step bootstrapping, a concept detailed by [55]. The Huber loss is defined as follows:

1 $$\begin{aligned} L_{\delta }(b_e, \theta ) = {\left\{ \begin{array}{ll} \frac{1}{2}b_e^2 &  \text {for } |b_e| \le \delta , \\ \delta (|b_e| - \frac{1}{2}\delta ) &  \text {otherwise}. \end{array}\right. } \end{aligned}$$

Here, $\delta$ represents a hyper-parameter set to 1 in our case, and $b_e$ denotes the temporal difference error related to a sample $e = \langle s, a, r\rangle$, which is calculated across multiple steps. The temporal difference for an *n*-step lookahead is as follows:

2 $$\begin{aligned} b_e = \left( \sum _{i=t}^{t+n-1} \gamma ^{i-t} R(s_i,a_i) + \gamma ^{n} \max _{a' \in A} \widehat{Q}(s_{t+n}, a', \theta ^\textsf{target}) \right) - \widehat{Q}(s, a, \theta ). \end{aligned}$$

This equation sums up rewards over *n* steps, beginning from the current step *t*, and applies a discount factor $\gamma ^{n}$ to the future *Q*-value, which is predicted using the target network with parameters $\theta ^\textsf{target}$. It then calculates the difference between this future *Q*-value and the estimated *Q*-value at the current state, $\widehat{Q}(s, a, \theta )$. Intuitively, the goal is to minimize this difference, aiming for precise *Q*-value estimations that reflect the true expected rewards. Let us note the use of a target network $\theta ^\textsf{target}$, as proposed by [52]. This architectural choice aims to diminish the correlations between the networks, fostering more stable updates. By updating the target network’s parameters less frequently, we ensure a more constant target over extended periods, which significantly improves the reliability of the learning signals.

Furthermore, employing an *n*-step lookahead strategy in reinforcement learning enhances the learning mechanism, especially in complex scenarios where the consequences of actions extend far into the future. This method, by incorporating a sequence of forthcoming rewards into its updates, provides a richer signal to the learning process. This leads to a quicker convergence of the estimated *Q*-values to their actual values. Additionally, the *n*-step method reduces the bias inherent in single-step updates by decreasing reliance on the immediately following state. Instead, it spreads the learning update over a sequence of future states. This approach results in a more comprehensive and balanced learning trajectory, significantly improving the robustness of the learning process.

Another standard mechanism is the $\epsilon$-greedy policy for action selection, providing a balance between exploration and exploitation. The key idea is to periodically perform a random action. The policy is defined as follows, where $\epsilon \in [0,1]$ is an hyper-parameter:

3 $$\begin{aligned} \pi (a|s) = {\left\{ \begin{array}{ll} \underset{a\in A}{\textsf{argmax}}\ \widehat{Q}(s,a,\theta ) &  \text {with probability } 1 - \epsilon , \\ a \sim _{\text {uniform}} A &  \text {with probability } \epsilon . \end{array}\right. } \end{aligned}$$

**Fig. 1:**
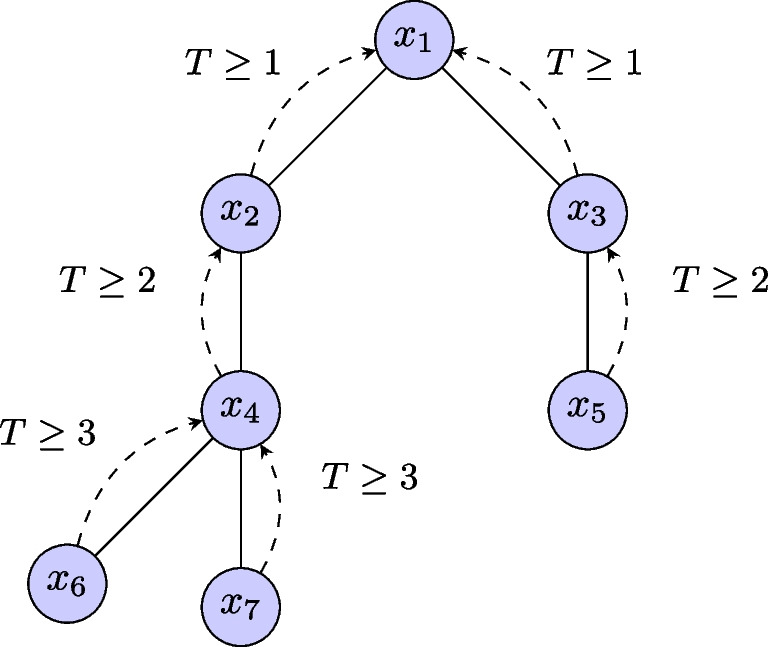
Information propagation in a graph neural network. After a single message passing step, node $x_1$ receives information from its direct neighbors ($x_2$ and $x_3$). After two steps, it will also have the information of its 2-hops neighbors ($x_4$ and $x_5$). After three steps, it will have the information of its 3-hops neighbors ($x_6$ and $x_7$). Such an operation is carried out for each node

A last component of our methodology is *experience replay* [52]. Consider a sample $e = \langle s, a, r \rangle$ that encapsulates an action *a* executed in state *s* and resulting in reward *r*. Every time an action is done during training, this sample is stored in a replay buffer $\mathcal {D}$. Subsequently, when updating the weights $\theta$, a mini-batch $\mathcal {B}$ consisting of randomly selected samples from $\mathcal {D}$ is utilized. The final loss calculation for these updates is described mathematically as follows:

4 $$\begin{aligned} \mathcal {L}(\mathcal {B}, \theta ) = \frac{1}{|\mathcal {B}|} \sum _{e\in \mathcal {B}} \Big [ L_{\delta }(b_e, \theta ) \Big ] \end{aligned}$$

Experience replay brings forth two significant advantages: first, it disrupts the correlation among sequential learning samples, thereby diminishing the variance in updates. Second, it boosts data efficiency through the reuse of past experiences across multiple updates. Then, we address the challenges posed by correlated data and the scarcity of diverse samples, thus elevating the efficacy of the deep *Q*-learning framework. The incorporation of these improvements improves the conventional deep *Q*-learning approach, aiming to forge a more resilient and efficient learning framework.

### Graph neural network

Intuitively, the goal of a *graph neural network* (GNN) is to embed information contained in a graph (e.g., the structure of the graph, spatial properties, local features of the nodes, etc.) into a task-specific *d*-dimensional embedding for each node $u \in V$ of the graph [56, 57]. To do so, information on a node is iteratively refined by aggregating information from neighboring nodes. Each iteration of aggregation is referred to as a *layer* of the GNN and involves parameters that can be learned depending on the downstream application. Let $h_u^{k} \in \mathbb {R}^{d\times 1}$ be the tensor representation of node *u* at layer *k* of the GNN, $h_u^{k+1} \in \mathbb {R}^{l \times 1}$ be the tensor representation of this node at the next layer (*l* being the dimension of a node at the layer $k+1$), and $\theta _1 \in \mathbb {R}^{l \times d}$ and $\theta _2 \in \mathbb {R}^{l \times d}$ be two matrices of parameters, respectively. Each GNN layer carries out the following update:

5 $$\begin{aligned} h_{u}^{k+1} = g\left( \theta _1 h_u^{k} ~ \star ~ \left( \bigoplus _{v \in N(u)} \theta _2 h_v^{k}\right) \right) ~ ~ \forall {u \in V}. \end{aligned}$$

Three operations are involved in this update: (1) $\bigoplus$ is an *aggregation* operator that is dedicated to aggregating the information of neighbors (e.g., *mean-pooling* or *sum-pooling*), (2) $\star$ is a *merging* which enables to combine of the information of a node with the ones from the neighbors (e.g., a concatenation), and (3) *g* is an element-wise non-linear activation function, such as the ones commonly used in fully-connected neural networks (e.g., $\textsc {ReLU}$ introduced by [58]). Without loss of generality, the *bias* term is not included in the equation. Through this operation, also known as *message passing*, features associated with a specific node are disseminated to its neighboring nodes. After *T* message-passing iterations, each node will have accumulated features from its *T*-hop neighbors. This dynamic is depicted in Fig. 1, with *T* representing the number of time-steps involved in the process. A concrete implementation of a GNN defines these three functions adequately, as we do later in our methodology. The training is conducted through back-propagation and an optimizer based on stochastic gradient descent such as Adam [59].

**Fig. 2:**
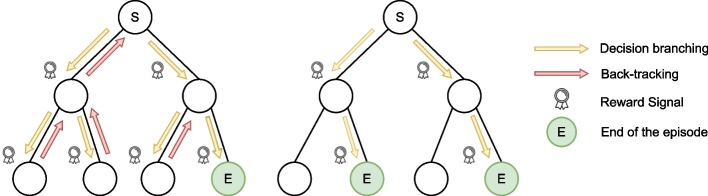
Visualization of the two training procedures. The left figure presents the *depth-first search* of [22] and the right figure presents our restart-based approach

## Learning value-selection heuristics inside a solver

This section presents how a value-selection heuristic can be learned with reinforcement learning in a CP solver from a model given as input. This is the core contribution of the paper. Three mechanisms are introduced: (1) *a training procedure based on restarts*, (2) *a reward function leveraging propagation of domains*, and (3) *a heterogeneous graph neural network architecture*. They are described individually in the next subsections. They have been implemented in the recently introduced *SeaPearl.jl* solver [22]. Inspired by the architecture of MiniCP [60], the main specificity of *SeaPearl* is to natively integrate support for learning inside the search procedure. This facilitates the prototyping of new search algorithms based on learning.

### Restart-based training

Generally speaking, the performance of a reinforcement learning agent is tightly correlated with the definition of an *episode*. This corresponds to the agent’s interactions with the CP solver’s search procedure and is related to the goal desired for the agent. Two options are discussed in this section, (1) an *episode based on depth-first search*, introduced by [22], and (2) an *episode based on restarts*, which is our first contribution.

#### Formalization of an episode

Building branching heuristics for solving exact combinatorial optimization problems often concurrently targets two objectives: *finding quickly good solutions* and *proving the optimality of a solution*. The approach of [22] relies heavily on the second objective and aims to minimize the number of visited search nodes before proving optimality (e.g., closing the search). To do so, they defined a training episode as a complete solving process carried out by the depth-first search of a solver and penalized through the reward function the generation of each node. This is illustrated in the left picture of Fig. 2. However, this approach suffers from an important difficulty. An episode only terminates when the search is completed, which is often intractable for realistic problems as it requires exploring an exponentially large search tree. This is especially problematic during training, where the heuristic is still mediocre. In addition, using a depth-first search algorithm in a *Markov Decision Process* (MDP) framework requires additional considerations not considered by [22]. For example, using a backtracking algorithm in a regular temporal MDP renders their method prone to the *credit assignment problem* [21]. These considerations have been pointed out by [19] for mixed-integer programming.

Unlike this approach, we propose to train the model to find high-quality solutions quickly. To do so, we followed the approach proposed by [5]: an episode is defined as a *single dive* in the search tree. No backtrack is allowed; the episode stops when a complete solution is found or when a failure results from the last branching decision. Once the episode is terminated, a restart from the root node is performed, and a new episode is generated, hence the name of *restart-based episode*. This is illustrated in the right picture of Fig. 2.

One limitation of [5] is that episodes are executed outside the CP solver during the training and cannot use the information updated during propagation for the branching. Inspired by [20] for variable-selection heuristics, we addressed this limitation by executing each episode inside the solver during the training. Formally, this requires defining the dynamics of the environment as a Markov Decision Process (i.e., a tuple $\langle S,A,T,R \rangle$, see Section 3.1). It is defined as follows.

*Set of states* Let $\mathcal {P} = \langle X, D(X), C, O \rangle$ be the expression of a combinatorial optimization problem (COP), defined by its variables (*X*), the related domains (*D*), its constraints (*C*), and an objective function (*O*). Each state $s_t \in S$ is defined as the pair $s_t = (\mathcal {P}_t,x_t)$, where $\mathcal {P}_t$ is a partially solved COP (i.e., some variables may have been assigned), and $x_t \in X$ is a variable selected for branching, at step *t* of the episode. The initial state $s_1 \in S$ corresponds to the situation after the execution of the fix-point at the root node. A terminal node is reached either if all the variables are assigned ($\forall x \in X: |D_t(x)| = 1$), or if a failure is detected ($\exists x \in X: |D_t(x)| = 0$). The variable selected for branching is obtained through any arbitrary heuristic such as the standard *first-fail* heuristic.

*Set of actions* Given a state $s_t = (\mathcal {P}_t,x_t)$, an action $a_t$ corresponds to the selection of a value $v \in D(x_t)$ for branching at step *t*. Finding the most promising value to branch on is the problem addressed in this paper.

*Transition function* Given a state $s_t = (\mathcal {P}_t,x_t)$ and an action $a_t=v$, the transition function executes three successive operations. First, it assigns the value *v* to the variable *x* (i.e., $D(x_{t+1}) = v$). Second, it executes the fix-point on $\mathcal {P}_t$ in order to prune the domains (i.e., $\mathcal {P}_{t+1} = \textsf{fixPoint}(P_{t})$). Third, it selects the next variable to branch on (i.e., $x_{t+1} = \textsf{nextVariable}(P_{t+1})$). This results in a new state $s_{t+1} = (\mathcal {P}_{t+1},x_{t+1})$. Integrating the propagation inside the transition is one important difference with [5].

*Reward function* The function is defined separately in Section 4.2.

#### Training algorithm

The training phase is summarized in Algorithm 1. It is mainly based on *deep Q-learning* and the principles described in Section 3.1. First, the model weights and the replay buffer are initialized (lines 9 to 11). The training is carried out for *I* iterations. At each iteration, an instance $\mathcal {P}$ is generated from an available generator $\mathcal {G^\mathcal {P}}$ of instances (line 13). Ideally, this generator should reflect the distribution of instances that need to be solved. Then, the MDP is built and the first state is initialized (lines 14 to 16). The nested loop consists in unrolling an episode until a terminal state (i.e., a feasible solution or a failure) is reached. The action to perform (i.e., the variable to assign) is selected thanks to an $\epsilon$-greedy policy (lines 18 to 22). The resulting reward and next state are subsequently inferred following the dynamics of the MDP (lines 23 to 24). We recall that all the classical operations of the solver (selecting the variable, executing the fix-point, etc.) are encapsulated in the transition function. The replay buffer is also updated with the new sample (line 25). When its size is exceeded, the oldest sample is removed from it. The weights are updated after each *K* iterations (lines 27 to 29). Similarly, the target network is updated at each *C* iterations (lines 31). Finally, the weights are returned when the training is over.

**Algorithm 1:**
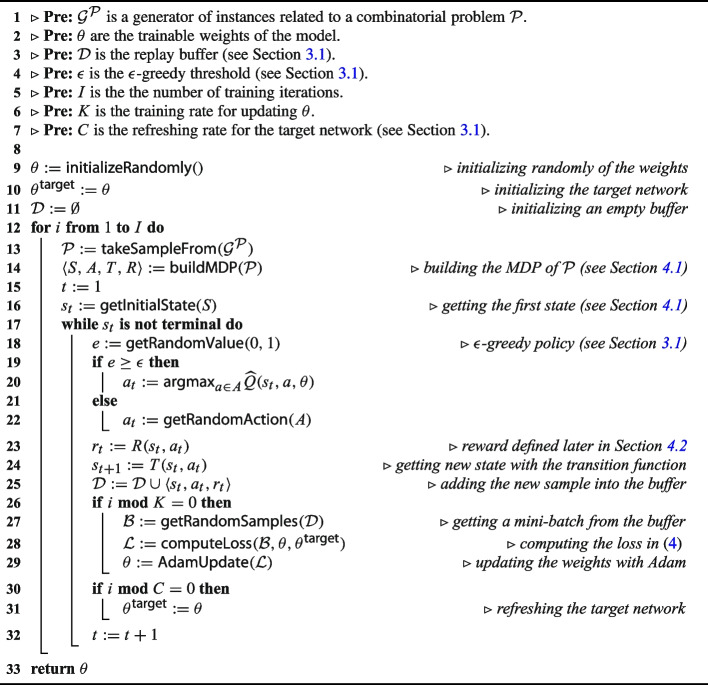
Overview of the training algorithm.

#### Comparison with depth-first search training

**Fig. 3:**
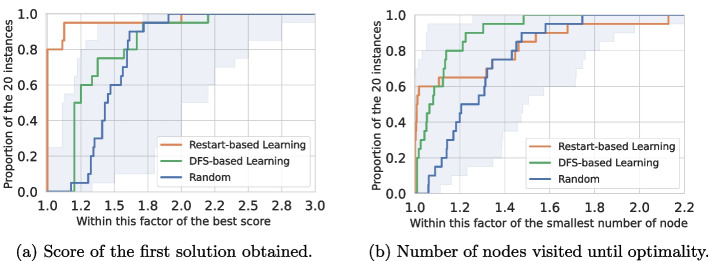
Comparison of both training methods on maximum independent set (50 nodes). As a non-learned baseline, we added the performances of an agent performing only random decisions. The shaded blue area corresponds to the worst and best values obtained with the random agent, across 10 trials per instance. Training is carried out on randomly generated Barabási-Albert graphs [62]; we selected this type of distribution as the generated graphs are known to mimic human-made and natural organizations. The evaluation is performed on 20 other graphs following the same distribution

We compared our restart-based training procedure using a simple terminal reward based on the solution’s score with the backtracking-based approach of [22] using their reward at each step (penalty of 1 for each explored node). We selected the *maximum independent set problem* for this comparison with instances with 50 nodes. Results are presented using performance profiles [61] in Fig. 3. A detailed explanation of the experimental protocol is proposed in Section 6.

We evaluated both methods on two metrics matching the objective for which they were specifically trained. We look at the value of the solution obtained after a single dive (Fig. 3a) in the tree search and the number of nodes visited to prove optimality using a depth-first search (Fig. 3b). As expected, we observe that the agent trained with the *restart-based learning* strategy allows good results regarding the optimality gap for the first solution found after a single dive. Remarkably, our method yields a comparable ability to prove optimality compared to [22], whose primary aim was specifically to solve the problem in the minimum number of nodes. This last result has to be mitigated as both RL-based methods lie in the range of the random strategy (shaded blue area).

Finally, as shown in Fig. 3a, it is important to notice that the optimality gap returned by our method is still non-negligible at the first solution obtained. The complexity of a combinatorial problem lies mainly in closing this gap, which is why backtracking during the solving phase is required. Experiments with backtracking are proposed in Section 6.

### Propagation-based reward

The definition of our reward must be aligned with our objective of *finding quickly good solutions* for the combinatorial problem. Based on our training procedure, an intuitive function is to reward the agent proportionally to the solution quality found at the end of an episode. In case of an infeasible solution found, a penalty can be given. The main drawback of this rewarding scheme is that this information is only available at terminal nodes, and no reward is provided in branching nodes. This is related to the *sparse reward* problem, which complicates the training process [63]. To address this challenge, one should find a way to give informative intermediate rewards along the solving process.

#### Formalization of the reward

**Fig. 4:**
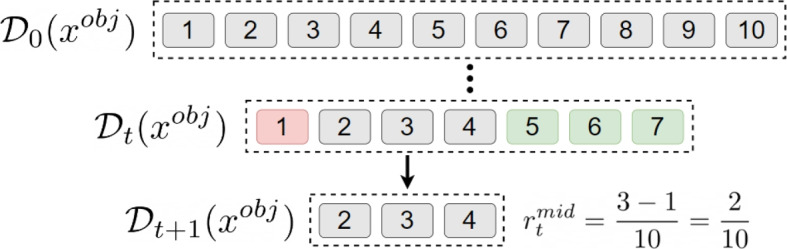
Intermediate reward when four values are pruned from the domain

We propose a new rewarding scheme based on the domain reduction of the objective variable (i.e., the variable that must be minimized or maximized). This reduction happens either thanks to the branching assignment or the application of the fix-point. There are two main components: (1) an *intermediate reward* ($r^\textsf{mid}$) collected at branching nodes, and (2) *terminal reward* ($r^\textsf{end}$) collected only at the end of an episode. Assuming a minimization problem, the intermediate reward follows two principles: each domain reduction of the largest values of the domain is rewarded, and each domain reduction of the lowest values of the domain is penalized. It is important to note that following these principles does not guarantee the discovery of a good solution at the end of the branch. The rationale is to lead the agent to a situation where the minimum cost can be *eventually* obtained while removing costly solutions. It is formalized in (6) to (8), where $r^\textsf{mid}_t$ is the reward obtained at step *t*, and is illustrated in Fig. 4.

As shown in (9), the terminal reward is set to -1 if the leaf node corresponds to an infeasible solution and 0 if it is feasible. Finally, the total reward ($r^\textsf{acc}$) accumulated during an episode of *T* steps is the sum of all intermediate rewards with the final term, as proposed in (10).

6 $$\begin{aligned} r^\textsf{ub}_t= &   \# \Big \{ v \in D_t(x^{\textsf{obj}}) ~ \Big |~ v \notin D_{t+1}(x^{\textsf{obj}}) \wedge v > \max \big (D_t(x^{\textsf{obj}})\big ) \Big \} \end{aligned}$$

7 $$\begin{aligned} r^\textsf{lb}_t= &   \# \Big \{ v \in D_t(x^{\textsf{obj}}) ~ \Big |~ v \notin D_{t+1}(x^{\textsf{obj}}) \wedge v < \min \big (D_t(x^{\textsf{obj}})\big ) \Big \} \end{aligned}$$

8 $$\begin{aligned} r^\textsf{mid}_t= &   \frac{r^\textsf{ub}_t - r^\textsf{lb}_t}{\big |D_1(x^{\textsf{obj}})\big |} \end{aligned}$$

9 $$\begin{aligned} r^\textsf{end}_t= &   - 1 \mathsf {~if~unfeasible~solution~found~} (0 \mathsf {~otherwise}) \end{aligned}$$

10 $$\begin{aligned} r^\textsf{acc}= &   \Big ( \sum _{t=1}^{T-1} r^\textsf{mid}_t \Big ) + r^\textsf{end}_T \end{aligned}$$

#### Comparison with the score reward

**Fig. 5:**
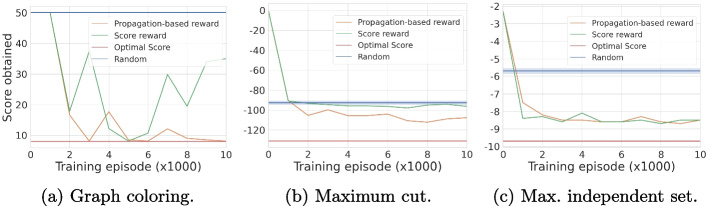
Training curve for the two rewarding schemes, each validation step corresponds to performing a single dive in the search tree, the *score* obtained refers to the quality of the solution found on the leaf node

An experimental analysis of this new reward scheme (*propagation-based reward*) is carried out for the *graph coloring*, *maximum cut*, and *maximum independent set* problems; we look at the quality of the solution found after a single dive in the search tree. As a baseline, we consider a reward (*score reward*) that only gives a value at terminal nodes ($r^\textsf{end}_T$) without an intermediate reward. Besides, we also consider the solutions returned by a random value-selection heuristic as a baseline. Figure 5 shows the evolution of the quality of the first solution returned (*y*-axis, averaged on 20 instances of the validation step) with the training time (number of episodes in the *x*-axis) using for training our restart-based search strategy defined in Section 4.1. Instances are Barabási-Albert randomly generated graphs with 50 nodes. Except for the rewarding scheme, the other parts of the architecture are unchanged. We observe that the *propagation-based reward* provides a more stable training (Fig. 5a) and can converge to a better model or, at least, to an equally good model as the terminal *score reward* (Fig. 5b and c).

**Fig. 6:**
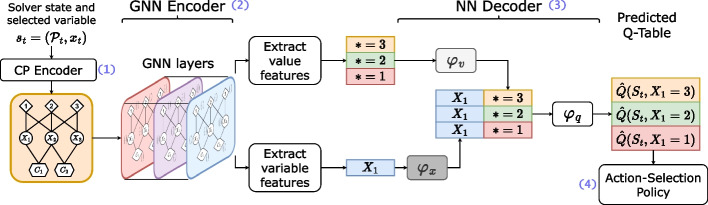
High-level overview of the neural architecture designed

It should be noted that depending on the problem, the reward signal may remain sparse inside episodes even with our definition; this explains the discrepancy across the three problems. Constraint propagation might take several steps to reach the objective variable, meaning that for related intermediate decisions, no value will be pruned from the domain of the objective variable. The *graph coloring* problem is thus the problem for which taking these intermediate rewards is the most beneficial. Indeed, any previously unused color added will negatively impact the domain of the objective function, yielding an insightful negative reward. Conversely, branching on the *maximum independent set* problem does not consistently impact the objective function domain through the mechanism of constraint propagation, particularly at the beginning of the search. Our method yields no worse result than the usual reward signal in this setting. This worst-case scenario empirically validates the robustness of this reward.

### Heterogeneous graph neural network architecture

An important part of the framework is the neural network architecture that we designed to perform a prediction of the next value to branch on. A high-level representation is proposed in Fig. 6. Four steps are carried out: (1) a *CP model encoder*, (2) a *graph neural network encoder*, (3) a *neural network decoder*, and (4) an *action-selection policy*. They are detailed in the next subsections.

#### CP Model encoder (step 1)

**Fig. 7:**
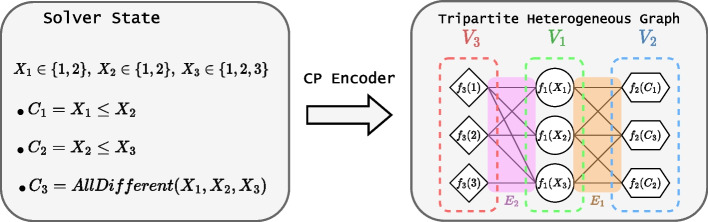
Representation computed by the CP encoder on a simple example

The core idea is to learn for any CP model given as input, unlike [5], who require a specific encoding for each combinatorial problem. This has been achieved for mixed-integer programs thanks to a bipartite graph representation [8] and by [22] for CP models thanks to a tripartite graph. This last work does not leverage any feature related to the variables, values, or constraints. We built upon this last approach by adding such features. Specifically, let $\mathcal {P} = \langle X, D(X), C, O \rangle$ be the combinatorial problem we want to encode. The idea consists in building a simple undirected graph $\mathcal {G}(V_1,V_2,V_3,f_1,f_2,f_3,E_1, E_2)$ encoding all the information of $\mathcal {P}_t$ from a state $s_t = (\mathcal {P}_t,x_t)$. In this representation, $V_1$, $V_2$, and $V_3$ are three sets of vertices, $f_1$, $f_2$, and $f_3$ are three sets of feature vectors, and $E_1$ with $E_2$ are two distinct sets of edges. This yields a graph with three types of nodes decorated with features. The first part of the encoding we propose is as follows: (1) each variable, constraint, and value corresponds to a specific type of node ($V_1 =X$, $V_2 = C$, and $V_3 = D$), (2) each time a variable $x \in V_1$ is involved in a constraint $c \in V_2$, an edge $(x,c) \in E_1$ is added between both nodes, (3) each time a value $v \in V_3$ is in the domain of a variable $x \in V_1$, an edge $(v,x) \in E_2$ is added between both nodes. This gives a tripartite graph representation of a CP model generically. This is illustrated in Fig. 7. The second part of the encoding is to add features to each node. Intuitively, the features will provide meaningful information and thus improve the quality of the model. The features we considered are proposed below. We note that we can easily extend this encoding by integrating new features.

1. *Features attached to variables (*$f_1$*)*: the current domain size, the initial domain size, a binary indication if the variable is already assigned, and a binary indication if the variable corresponds to the objective.
2. *Features attached to constraints (*$f_2$*)*: the constraint type (one-hot encoding), and a binary indication if the constraint propagation has reduced domains.
3. *Features attached to values (*$f_3$*)*: its numerical value.

#### Graph neural network encoder (step 2)

Once the CP model has been encoded as a graph, the next step is to embed this representation as a latent vector of features for each node of the graph (see Section 3.2). We propose to carry out this operation with a graph neural network. Unlike the standard prediction scheme presented in (5), our graph has three types of nodes. For this reason, we opted for a *heterogeneous* architecture. Concretely, a specific convolution is carried out for each node type. The architecture is detailed in (11) to (13), where $\bigoplus$ is the *sum-pooling* or *mean-pooling* aggregation, operator $(.\Vert .)$ is a concatenation of vectors, $N_x(n)$ is the set of neighbouring nodes of *n* from $V_1$ (variable), $N_c(n)$ is the set of neighbouring nodes of *n* from $V_2$ (constraint), $N_v(n)$ is the set of neighbouring nodes of *n* from $V_3$ (value), $\theta _{1,\dots ,10}^k$ are weight matrices at layer *k*, and *g* is the leakyReLU activation function [64]. Another difference with the canonical GNN equation is the integration of *skip connections* ($h_x^{0}$, $h_c^{0}$, and $h_v^{0}$) allowing to keep at each layer information from the input features. This technique is ubiquitous in deep convolutional networks such as in *ResNet* [65]. Finally, the initial embedding is initialized as follows: $h_x^{0} = \theta _{11} f_1$, $h_c^{0} = \theta _{12} f_2$, and $h_v^{0} = \theta _{13} f_3$, where $\theta _{11,\dots ,13}$ are new weight matrices.

11 $$\begin{aligned} h_{x}^{k+1}= &   g \Big (\theta _1^k h_x^{0} ~ \big \Vert ~ \theta _2^k h_x^{k} ~ \big \Vert ~ (\bigoplus _{c \in N_c(x)} \theta _3^k h_c^{k}) ~ \big \Vert ~ (\bigoplus _{v \in N_v(x)} \theta _4^k h_v^{k}) \Big ) \forall {x \in V_1}\end{aligned}$$

12 $$\begin{aligned} h_{c}^{k+1}= &   g \Big (\theta _5^k h_c^{0} ~ \big \Vert ~ \theta _6^k h_c^{k} ~ \big \Vert ~ (\bigoplus _{x \in N_x(c)} \theta _7^k h_x^{k}) \Big ) \forall {c \in V_2} \end{aligned}$$

13 $$\begin{aligned} h_{v}^{k+1}= &   g \Big (\theta _8^k h_v^{0} ~ \big \Vert ~ \theta _9^k h_v^{k} ~ \big \Vert ~ (\bigoplus _{x \in N_x(v)} \theta _{10}^k h_x^{k}) \Big ) \forall {v \in V_3} \end{aligned}$$

#### Neural network decoder (step 3)

At this step, a *d*-dimensional tensor is obtained for each graph node. Let $x \in V_1$ be the node representing the current variable selected for branching, and $V_x \subseteq V_3$ the subset of nodes representing the values available for *x* (i.e., the values that are in the domain of the variable). The goal of the *decoder* is to predict a *Q-value* (see Section 3.1) for each $v \in V_x$. The computation is formalized in (14), where $h_{x}^{K}$ and $h_{v}^{K}$ are the node embedding of variable *x* and value *v*, respectively, after *K* iterations of the GNN architecture. The functions $\varphi _x: \mathbb {R}^{d} \rightarrow \mathbb {R}^l, \varphi _v: \mathbb {R}^{d} \rightarrow \mathbb {R}^l, \varphi _q: \mathbb {R}^{2l} \rightarrow \mathbb {R}$ are fully-connected neural networks. Such a *Q*-value must be computed for each value $v \in V_x$. It is internally done thanks to matrix operations, allowing a more efficient computation.

14 $$\begin{aligned} \widehat{Q}(h_{x}^{K}, h_{v}^{K}) = \varphi _q \Big ( \varphi _x(h_{x}^{K}) ~ \big \Vert ~ \varphi _v( h_{v}^{K}) \Big ) ~ ~ \forall v \in V_x \end{aligned}$$

#### Action-selection policy (step 4)

Once all the *Q*-values have been computed for the current variable, the policy is defined by an *explorer* that can decide to exploit the approximated *Q*-values by greedily choosing the best action as shown in (15) or decide to select unpromising action associated with a lower *Q*-value (for example, by selecting a random action with a $\epsilon$-greedy policy). This behavior derives from the trade-off between exploitation and exploration, which is necessary for early learning when the estimates of Q-values are poor, and when only a few states have been visited. Once trained, the *Q*-values should represent the branching choice leading to the best decision according to the reward of (10).

15 $$\begin{aligned} \pi (v | x) = \textsf{argmax}_{v \in V_x} \hat{Q}(h_{x}^{K}, h_{v}^{K}) \end{aligned}$$

By integrating all these elements, the architecture provides a data-driven value-selection heuristic within a constraint programming solver.

## Solving algorithm

This section presents how the value-selection heuristic designed in Section 4 can be used inside a CP solver for solving new problems. First, we opted to embed our predictions inside an *iterative limited discrepancy search* (ILDS) [66]. We highlight that this strategy is different from the *restart-based* one used for training. Iterative limited discrepancy search is commonly used when we are confident in the quality of the heuristic. The core idea is to restrict the number of branching choices deviating from the heuristic (i.e., a *discrepancy*). By doing so, the search will explore a subset of solutions expected to be good while giving a chance to reconsider the value-heuristic selection which is nevertheless prone to errors. This mechanism is enriched with a procedure that iteratively increases the number of discrepancies allowed once a level has been explored.

The search procedure is depicted in Algorithm 2. It takes as input the combinatorial problem to solve ($\mathcal {P}$), the weights learned in Algorithm 1 ($\theta$), the graph neural network outputting a *Q*-value for each value ($\widehat{Q}$), and the number of iterations for the ILDS (*I*). For each number *i* of discrepancies allowed, a new search $\Psi$ is initialized and executed on $\mathcal {P}$ (line 8). Until the search is not completed, the following operations are carried out: executing the fix-point on the current node (line 10), selecting the next variable *x* to branch on (line 11), getting the related state (line 12), encoding it with the MDP definition (line 13), taking the most promising value *v* thanks to the learned model (line 14), and branching on the assignation $x=v$ (line 15). Line 15 is also responsible to restart the search when a terminal node is reached, as commonly done in LDS. Finally, the minimum cost solution is tracked (line 16) and returned (line 17).

**Algorithm 2:**
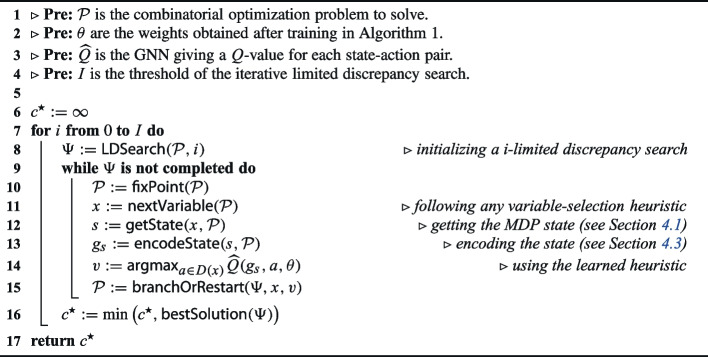
Integrating the learned heuristic inside a CP solver.

## Experiments

The goal of this section is to evaluate the quality of the learned value-selection heuristic and the efficiency of the approach. Four combinatorial optimization problems are considered: *graph coloring* (COL), *maximum independent set* (MIS), *maximum cut* (MAXCUT), and *minimum vertex cover* (MVC).

### Experimental protocol

Three configurations for the distribution of the problems generated are proposed for each problem: *small* (20 to 30 nodes), *medium* (40 to 50 nodes), and *large* (80 to 100 nodes) instances. Training is carried out on randomly generated Barabási-Albert graph [62] with a density factor varying between 4 and 15 according to the size of the instances. A specific model is trained for each configuration of each combinatorial problem. The training is done using randomly generated instances. Evaluation is then performed on 20 new graphs following the same distributions. The models are trained on an Nvidia Tesla V100 32Go GPU until convergence. It took up to 72 hours of training time for the most difficult cases (*graph coloring* with 80 nodes) and less than 1 hour for the simplest cases (*graph coloring* with 20 nodes). Each operation of the CP solver during training and evaluation is carried out on a CPU Intel Xeon Silver 4116 at 2.10GHz. The approach has been implemented in *Julia* and is integrated into the solver *Seapearl*. The implementation is available on GitHub with BSD 3-Clause licence.^2^

We compared our approach (Learned, ILDS) with two other generic value selection heuristics: *impact-based search* (Impact) [2] and *activity-based search* (Activity) [3]. The standard *minDomain* heuristic is used for the variable selection. Comparisons with [22] have been provided in Section 4.1. As it has been highlighted that this approach is not suited to find good solutions quickly, it is not included again in the next experiments. Each approach is evaluated with a fixed node budget depending on the parameters of the distribution used to generate the problems. For our approach, the performance obtained after the first dive in the tree search is also monitored (Learned, 1st dive). As Impact and Activity are online learning methods, they perform similarly to a random selection at the beginning of the search. For this reason, the performance obtained after the first dive in the tree search with such methods is omitted. Finally, we also included a comparison with a random selection using DFS with the same node budget (Random). Finally, the optimal cost (OPT) has been obtained with an exact approach without any restriction on the budget.

### Results: performance of the learned heuristics

Table 1 summarizes the main results of our approach. As a general comment, our approach can find solutions of superior quality given a node budget or find the optimal solution by exploring fewer nodes than the baselines. Interestingly, our approach (Learned, ILDS) can learn a branching strategy giving high-quality solutions, even without backtracking (1st dive). For instance, a single dive for *maximum cut* with 50 nodes yields almost instantly a solution with an optimality gap of 0.16, whereas a depth-first search with a random selection (Random, DFS) required 19 seconds and roughly 53,000 nodes explored to find a solution with a similar gap. Within this same budget, (Learned, ILDS) significantly improves the solution and achieves an optimality gap of 0.09. It is worth highlighting that (Learned, ILDS) took 130 seconds to explore 38,744 nodes and has, thereby, an exploration rate slower than the other methods. This significantly increased execution time is mainly because calling the graph neural network architecture (Section 4.3) at each tree search node is much more computationally expensive than calling a simple heuristic. This difficulty is further discussed in Section 6.5.

**Table 1:** Results for the three problems given a fixed node budget ($\mathcal {B}$) related to each configuration

	Graph coloring problem (COL)								
	20 nodes ($\mathcal {B}= 10^3$)			40 nodes ($\mathcal {B}= 10^4$)			80 nodes ($\mathcal {B}= 10^5$)		
	Gap	Node	Time	Gap	Node	Time	Gap	Node	Time
Random DFS	0.00	378	$$ < 1$$	0.00	1,735	$$ < 1$$	0.00	7,211	2
Activity-based DFS	0.00	378	$$ < 1$$	0.00	1,664	$$ < 1$$	0.00	7,051	2
Impact-based DFS	0.00	374	$$ < 1$$	0.00	1,732	$$ < 1$$	0.00	7,057	2
Learned 1st dive	0.06	−	$$< 1$$	0.08	−	$$ < 1$$	0.06	−	$$ < 1$$
Learned ILDS	0.00	27	$$ < 1$$	0.00	104	$$ < 1$$	0.00	120	$$ < 1$$
	Maximum independent set problem (MIS)								
	30 nodes ($\mathcal {B}= 10^3$)			50 nodes ($\mathcal {B}= 10^4$)			100 nodes ($\mathcal {B}= 10^5$)		
	Gap	Node	Time	Gap	Node	Time	Gap	Node	Time
Random DFS	0.00	293	$$ < 1$$	0.00	8942	1	0.10	41,774	9
Activity-based DFS	0.00	215	$$ < 1$$	0.00	5807	1	0.09	35,536	7
Impact-based DFS	0.00	297	$$ < 1$$	0.00	7474	1	0.10	38,154	8
Learned 1st dive	0.08	−	$$ < 1$$	0.09	−	$$ < 1$$	0.20	−	$$ < 1$$
Learned ILDS	0.00	88	$$ < 1$$	0.00	539	1	0.02	28,392	253
	Minimum vertex cover problem (MVC)								
	30 nodes ($\mathcal {B}= 10^4$)			50 nodes ($\mathcal {B}= 10^5$)			100 nodes ($\mathcal {B}= 10^5$)		
	Gap	Node	Time	Gap	Node	Time	Gap	Node	Time
Random DFS	0.00	253	$<1$	0.00	6,334	1	0.03	48,505	11
Activity-based DFS	0.00	359	$<1$	0.00	7,684	1	0.04	36,967	9
Impact-based DFS	0.00	369	$<1$	0.00	7,583	1	0.04	48,505	8
Learned 1st dive	0.05	−	$$ < 1$$	0.04	−	$$ < 1$$	0.07	−	$$ < 1$$
Learned ILDS	0.00	44	$<1$	0.00	1,189	3	0.01	24,037	207
	Maximum cut problem (MAXCUT)								
	20 nodes ($\mathcal {B}= 10^4$)			50 nodes ($\mathcal {B}= 10^5$)			80 nodes ($\mathcal {B}= 10^5$)		
	Gap	Node	Time	Gap	Node	Time	Gap	Node	Time
Random DFS	0.04	4,877	1	0.17	53,110	19	0.23	50,424	25
Activity-based DFS	0.04	4,635	1	0.17	44,664	14	0.21	66,791	33
Impact-based DFS	0.03	5,959	2	0.17	47,970	17	0.21	64,602	32
Without fine-tuning									
Learned 1st dive	0.15	−	$$ < 1$$	0.16	−	$$ < 1$$	0.33	−	$$ < 1$$
Learned ILDS	0.03	3,714	5	0.09	38,744	130	0.23	34,843	279
With fine-tuning									
Learned 1st dive	−	−	−	−	−	−	0.09	−	$$ < 1$$
Learned ILDS	−	−	−	−	−	−	0.05	61,872	625

The average result (rounded) on the 20 test instances is reported for each configuration. *Gap* indicates the optimality gap, *Node* gives the number of nodes explored before finding the best solution within the budget, and *Time* gives the time (seconds) before finding this solution

**Fig. 8:**
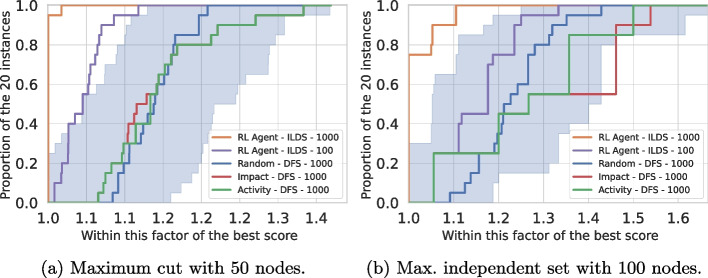
Best solutions found within a restricted node budget on largest instances for the three problems considered. We set a small budget to evaluate the ability of each approach to *find quickly a good solution*, which is the objective aimed by this work. The performance profile ratio is computed using the optimal solution as a reference. Within the same maximal number of nodes visited (1000), we observe that (Learned, ILDS) dominate all the other methods. Besides, we still perform better than the baselines when restricting ten times the budget for (Learned, ILDS)

Concerning Activity and Impact heuristics, they yield no improvement on *graph coloring* compared to a random strategy. This can be explained by the fact that this class of problem has many possible combinations of variables and values for branching. This requires a significantly larger number of explored nodes to initialize these two heuristics efficiently. For the three other problems, characterized by a binary domain for the values to branch on, Activity and Impact provide significantly better results than the random strategy, which is the expected behavior. Interestingly, (Learned, ILDS) provides the best optimality gap within the node budget for all the situations tested, except for *maximum cut* with 80 nodes, without fine-tuning. We anticipate that a larger model may be necessary to address this situation or that the training time was not enough. The benefits of fine-tuning to tackle this challenge are discussed in Section 6.4. Additional results are proposed in Fig. 8 using performance profiles [61] for two hard situations (100 for *maximum independent set*, and 50 for *maximum cut*) given a node budget of 100 or 1000 nodes.

**Fig. 9:**
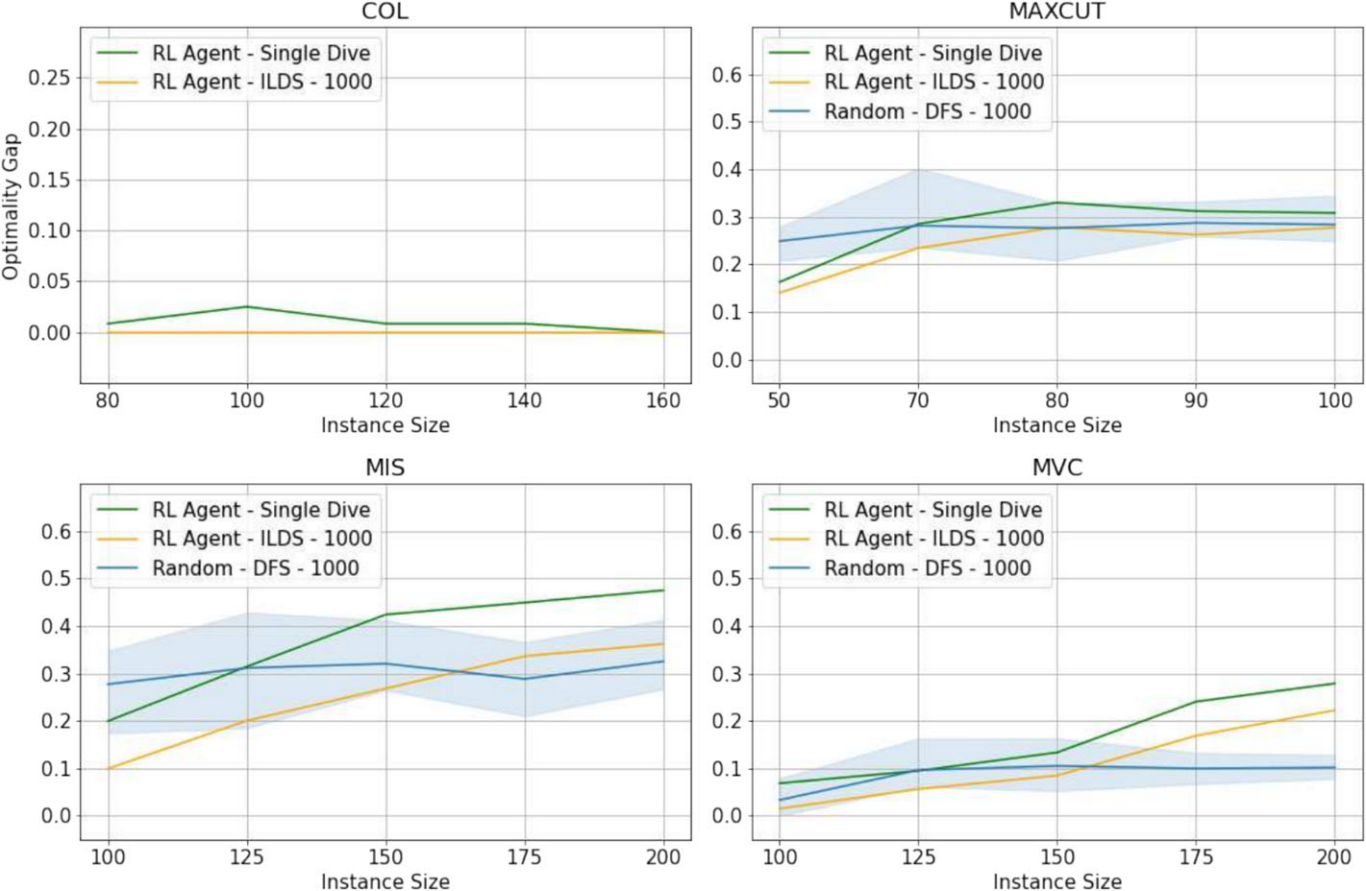
Analysis of the generalization ability on larger instances. *Graph coloring* is on the top-left, *maximum cut* on the top-right, *maximum independent set* on the bottom-left, and *minimum vertex cover* on the bottom-right. Each plot presents three curves: the performance of the learned heuristic with a single dive, the performance of the learned heuristic with a budget of 1,000 nodes with ILDS, and the performance with of the random heuristic a budget of 1,000 nodes with DFS

### Analysis: generalization to larger instances

Figure 9 illustrates the generalization capabilities of the learned heuristic across 20 new instances of increasing sizes, without necessitating retraining. The heuristic maintains commendable performance on instances whose sizes are akin to those encountered during training (i.e., at most 25 additional nodes). This observation suggests that incorporating a broader range of instance sizes and a greater diversity of nodes during the training phase could further enhance performance.

However, there is a noticeable performance decline in solving *maximum cut*,*maximum independent set*, and *minimum vertex cover* problems for the strategies (Learned, 1st dive) and (Learned, ILDS). Specifically, as the problem size increases, the performance gap aligns more closely with the random heuristic. Contrastingly, the model trained on *graph coloring* displays remarkable robustness, consistently achieving the optimal solution for instances with a budget of 1,000 nodes, regardless of size variation, up to 160 variables. This stands in contrast to the random heuristic, which consistently fails to identify high-quality solutions within the same computational budget (gap exceeding the threshold on the *y*-axis).

### Analysis: handling OOD instances with fine-tuning

**Fig. 10:**
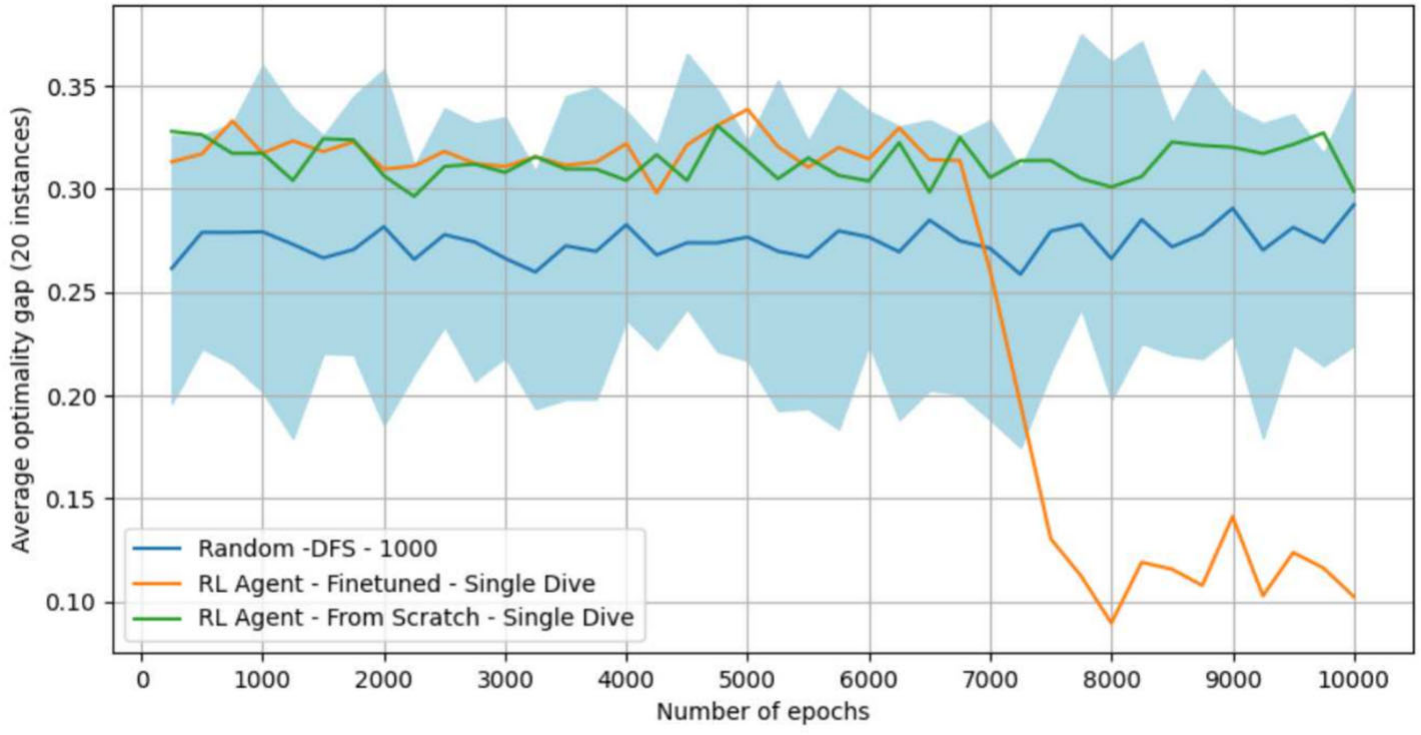
Analysis of our approach’s fine-tuning ability. The *x*-axis represents the number of epochs used for fine-tuning on *maximum cut* instances (80 nodes), and the *y*-axis shows the optimality gap of the models on 20 *maximum cut* instances

This experiment focuses on the *maximum cut* problem with 80 nodes, where the model initially failed to outperform baselines (Table 1), likely due to time budget limitations in training. To address this, we applied *fine-tuning*, starting from a model trained on *maximum independent set* instances (100 nodes) and fine-tuning it on *maximum cut* instances (80 nodes) for up to 10,000 epochs (approximately 30 hours). Figure 10 shows the results for: (1) a model trained from scratch on *maximum cut*, (2) a model trained on *maximum independent set* and fine-tuned on *maximum cut*, and (3) the random baseline. The fine-tuned model significantly outperforms the one trained from scratch within the same training budget and is now competitive with the baselines, which was not the case for the vanilla model. This experiment highlights the benefits of fine-tuning for handling out-of-distribution instances with a lower training time budget.

### Discussions and opportunities of further research

The previous experiments showcased the promise of this framework to quickly find good solutions towards a generic value-selection heuristic inside a CP solver. There are nonetheless open challenges that must be considered for practical use. Six of them are discussed. It is worth noting that several of these challenges overlap with those identified by [48] for the broader application of graph neural networks in combinatorial optimization.

#### Challenge 1: scalability of the representation

Our approach faces a double penalty regarding its *scaling* capability: as the problem grows larger, the tripartite representation increases significantly in size, which results in a longer computation time required to make one branching decision. This impacts both training and evaluation. Additionally, the number of nodes (and, therefore, decisions to be made) in the search tree grows exponentially with the problem size, exacerbating the aforementioned phenomenon. This issue has also been observed by [24]. Consequently, our approach is penalized twice due to the exponential behavior of combinatorial problems. As a concrete example, *graph coloring* instances with 80 nodes require 72 hours of training on a GPU, while only 1 hour is required for the smallest instances. An interesting research direction to mitigate this difficulty is to build a mechanism to compact the representation, for instance, thanks to network pruning tools [67] or with *transfer learning*. Another idea is to call the model only in a few nodes, in a similar fashion as [12] did for *decision-diagram-based branch-and-bound* [68]. Analyzing how the *lottery hypothesis* could be applied in our situation is also an interesting direction [69]. On a lower level of computation, standard constraint programming solvers perform sequential decisions and are therefore optimized for CPU architecture. Concerning the training, it is carried out on a GPU. In the current implementation, each branching decision requires loading the entire tripartite graph on the Video RAM, which is inefficient. We believe much work could be done to optimize this CPU/GPU architecture, for instance by delegating other operations on the GPUs, such as the propagation of few constraints [70–72].

#### Challenge 2: tackling highly constrained problems

The experiments proposed in the paper considered combinatorial problems where the difficulty lay in finding the *best* solution. Still, it was easy to find a *feasible* solution, even of poor quality. We empirically observed that the learning performance largely depends on the abundance of feasible solutions in the search space. This is explained by the definition of the reward, which is based on the propagation occurring on the objective variable (see $r_t^{\textrm{mid}}$ in Section 4.2). However, when feasible solutions are not easily obtained, such as in highly constrained problems, the reward signal becomes less informative. Addressing such combinatorial problems remains an open challenge. We believe an extension of the reward signal can address this in order to handle other situations.

#### Challenge 3: learning a combined variable/value heuristic

Although this work proposes to learn a value-selection heuristic, learning how to branch on variables has already been considered in the literature [20]. An interesting research direction is to adapt this architecture to learn a variable-selection and a value-selection heuristic in a unified way. A possible direction is to consider a model with a double-head decoder, the first for selecting the variable and the second for selecting the value. On the training aspect, two reinforcement learning agents could be trained, with an the incentive to cooperate with the information sharing [73].

#### Challenge 4: proving the optimality of a solution

The goal pursued in this paper is to find the best solution as quickly as possible. Another direction is to guide the search to speed-up the optimality proof. It is what has been proposed by [22]. In practice, finding good solutions and proving optimality are complementary aspects inside a constraint programming solver and should be both considered. Possible directions to do so could be to redefine the reward function appropriately or to revise the definition of an episode, as proposed by [19] with TreeMDPs.

#### Challenge 5: handling out-of-distributions instances

Section 6.3 underscored the challenge of generalizing to instances that diverge from the distribution encountered during training, a well-documented issue in the field of machine learning research [74]. Addressing this challenge is pivotal for the practical application and deployment of machine learning models. *Fine-tuning* methods, as explored in Section 6.4, have shown success in various learning tasks [75, 76] and offer a promising solution to this challenge [77].

#### Challenge 6: selecting an appropriate graph neural network

Beyond issues of generalization, graph neural networks face inherent computational limitations. [78] demonstrated that the ability of any GNN architecture to differentiate between non-isomorphic graphs is constrained by the capabilities of the 1-dimensional *Weisfeiler-Leman* algorithm. This polynomial-time heuristic for the graph isomorphism problem has notable limitations, such as its failure to recognize cyclic information or to differentiate non-isomorphic bipartite graphs effectively [79]. In the context of branching for mixed-integer programming, [80] pinpointed a critical limitation concerning the expressive power of graph neural networks. Specifically, they highlighted that there exist instances with different strong branching scores that cannot be distinguished by any GNN based on message passing (as ours), irrespective of the network’s parameter count. This discovery underscores the necessity of exploring suitable architectures for branching, marking it as a compelling avenue for future research.

## Conclusion

The efficiency of constraint programming solvers is partially due to the branching heuristics used to guide the search. In practice, value-selection heuristics are often designed thanks to problem-specific expert knowledge, often out of reach for non-practitioners. In this paper, we proposed a method based on reinforcement learning for obtaining such a heuristic, thanks to historical data, characterized by problem instances following the same distribution of the one that must be solved. This has been achieved thanks to a restart-based training procedure, a non-sparse reward signal, and a heterogeneous graph neural network architecture. Experiments on four combinatorial optimization problems show that the framework can find better solutions close to optimality in fewer nodes visited than other generic baselines. Several limitations and challenges (e.g., tractability for larger or real-world instances, transfer learning, sparsity of the reward signal) have been identified, and addressing them is part of future work. We also plan to consider other combinatorial problems, such as the ones proposed in XCSP3 competitions [81].

## Footnotes

1 https://www.minizinc.org/doc-2.7.0/en

2 https://github.com/corail-research/SeaPearl.jl
